# Food safety knowledge, attitude, and practice among male and female food handlers: Evidence from fruit and vegetable producers in Ethiopia

**DOI:** 10.1016/j.heliyon.2023.e17301

**Published:** 2023-06-17

**Authors:** Girma Gezimu Gebre, Tibebu Legesse, Asmiro Abeje Fikadu

**Affiliations:** a The Japan Society for the Promotion of Science (JSPS) Postdoctoral Research Fellowship Program, Ritsumeikan University, Kyoto, 603-8577, Japan; b Departments of Agribusiness and Value Chain Management, College of Agriculture, Hawassa University, Hawassa, Ethiopia; c Department of Agricultural Economics, Debre Tabor University, Debre Tabor, Ethiopia; d Department of Agricultural and Resource Economics, Graduate School of Bioresource and Bioenvironmental Sciences, Kyushu University, Fukuoka, Japan

**Keywords:** Attitude, Food safety, Gender, Knowledge, Practice, Ethiopia

## Abstract

Studies on the level of knowledge, attitude and handling practice towards food safety would help to determine the associated factors of knowledge, attitude and practice towards food safety; however, these studies did not explicitly address food safety concerns related to fruit and vegetables in Ethiopia. Men and women could have different levels of knowledge, attitude, and handling practice; however, these gendered effects were not addressed in previous studies. Using data collected in 2021 from 311 farm households in Ethiopia, this study aimed to analyze the level of knowledge, attitude, and handling practice of fruit and vegetable and associated factors among male and female food handlers. Data was analyzed using descriptive statistics, spearman's correlation, and econometric models (logit, multinomial logit and ordered probit). Education has a positive significant effect on knowledge, attitude and handling practices of fruit and vegetable handlers. However, the effect was higher in the females. No correlation could be obtained between knowledge and practices for the total respondents. However, knowledge had a positive but weak significant association with practices in the female group. We found a significant positive correlation between knowledge and attitude. These findings indicated that food safety knowledge of fruit and vegetable handlers will influence their attitude and a positive attitude would influence the practice in safe handling of fruit and vegetables. It therefore may require more targeted campaigns (i.e. from awareness creation to behavior change) to increase the ability of the community members to adopt best practices while reducing the barriers associated with consuming unhealthy diets.

## 1 Introduction

Foodborne diseases cause a million cases of illness and thousands of deaths and are regarded as a public health problem in both developed and developing countries [1,2]. The most common causes of food-borne diseases include inadequate cooking, insufficient heating, long time between food preparation and consumption, improper storage, inadequate washing of equipment, and contaminated raw materials [[3], [4], [5]] and poor educational status [6]. According to Ref. [7] about 600 million cases and 420,000 deaths are reported on an annual basis, associated with foodborne disease. The WHO further noted that the rate of foodborne diseases has been on the rise in recent years [9]; however, the adverse effects are more on the health and economy of developing countries than their developed counterparts. Annually, more than one-third of the world's developing population are infected with food-borne diseases [9], which can be prevented by maintaining food safety practices [8,[10], [11], [12]].

In Ethiopia, around 70% of diarrheal disease is associated with the consumption of contaminated food [13]. Approximately 10–20% of food-borne disease outbreaks are due to contamination by the food handler [14]. The Federal Ministry of Health of Ethiopia acknowledges the depth of the problem by stating that communicable diseases such as diarrheal and intestinal parasites are the leading causes for outpatient attendance and causes of hospitalization, most of which are attributed to poor food safety*.* Furthermore, many reported cases of food-borne viral diseases have been attributed to infected food-handlers involved in catering services [15]. Therefore, food safety is a critical issue, particularly, for countries such as Ethiopia [4,16], which includes the control of physical, chemical, and biological hazards of food from farm-to-table [17]. According to Ref. [8], food safety is defined as the conditions and measures that are necessary during the production, processing, storage, distribution, and preparation of food to ensure that it is safe, sound, and wholesome, and fit for human consumption [15].

Studies on the level knowledge, attitude and handling practices towards food safety could enable regulatory authorities to take evidence derived measures on the consumption of safe food [9,[18], [19], [20], [21], [22], [23], [24]]. Such studies would help to determine the associated factors of knowledge, attitude and practices towards food safety. In Ethiopia, a few studies [[25], [26], [27]] were conducted on food safety knowledge, attitude, and practices of food handlers. These studies did not explicitly address food safety concerns related to fruit and vegetables. Even though fruit and vegetable consumption is crucial to the availability of micronutrients to the human body [[28], [29], [30]], the level of knowledge, attitude and handling practices towards the safety of fruit and vegetables and associated factors is not yet studied in Ethiopia.

On the other hand, because of gendered social norms and traditions, men and women participating in fruit and vegetable production, processing, distribution, and consumption could have different levels of knowledge, attitude, and handling practices [31]. The past study showed that being female and having a high education were associated with increased consumption of fruit and vegetables. However, the direction and strength of these relationships depends on gender and regional affiliations [32]. The relationship between women's education and fruit and vegetable safety (through production to consumption) is not clear in Ethiopia. Thus, this study aimed to determine the level of knowledge, attitude and practice towards the food safety of fruit and vegetables and associated factors among male and female food handlers in rural Ethiopia.

## 2 Materials and methods

### 2.1 The study area

**Fig. 1:**
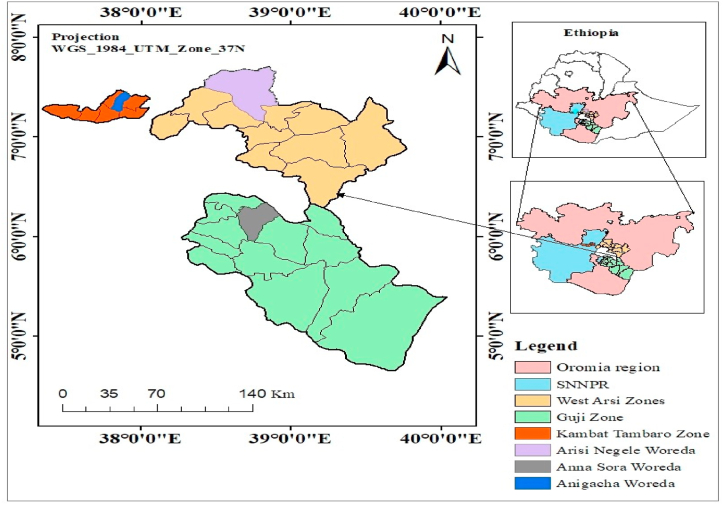
Location of the study area, Source: Authors.

The study was undertaken in three woredas in two regional states in Ethiopia: South Nation Nationalities and People (SNNP) region and Oromia region. Angecha woreda in the SNNP region and Anna Sora and Arsi Negele Woredas in the Oromia region. Angecha has a total population of 88,083, of whom 44,026 are women and 44,057 men. Anna Sora and Arsi Negele woredas are located in the Guji and Arsi Negele zones of the Oromia region, respectively (see Fig. 1). The agroecology of the Ana Sora woreda is 27% highland, 70% midland, and 3% lowland. This woreda is characterized by mixed economic activities, mainly agricultural practices which constitute the major livelihoods of the people [33,34]. A survey of the land in Arsi Negele woreda shows that 29.9% is cultivable, 4.3% pasture, 5.2% forest, and the remaining 60.6% is considered swampy, degraded or otherwise unusable. According to the 2007 national census report, the total population of Arsi Negele woreda was 260,129, of whom 128,885 were men and 131,244 were women [35].

### 2.2 Data collection methods

A multistage sampling procedure was used to collect field data in December 2021. First, three zones were selected from the SNNP and the Oromia regions. Second, three woredas were selected from three zones (one from each zone) based on their vegetable and fruit production potential. Third, from each woreda, two kebeles were selected (a total of six kebeles) based on availability of vegetable and fruit farms. Fourth, on average, 51 to 54 vegetable and fruit producer households were randomly selected from each kebele based on probability proportional to size. A total of 311 vegetable and fruit producing households were selected. From each household, one respondent was randomly selected and interviewed (see Table 1); 185 males, 126 females. The sample size was calculated using the Fisher [36] formula. Data was collected using semi-structured questionnaires. Participation in the interview was voluntarily based. Before interviews took place, the selected participants were informed about the purpose of the study and given the opportunity to refuse upon understanding the purpose.

**Table 1:** Structures and number of the sample respondents.

Region	Zone	Woreda	Kebelle	Number of sampled respondents		
				Male	Female	Total
**Oromia**	Guji	Anna sora	Ababo Kobo	37	14	51
			Raya Boda	35	15	50
	West Arsi	Arsi Negelle	Gubete Arjo	20	31	51
			Turge Gallo	30	21	51
**SNNPR**	Kambata Tambaro	Angecha	Kerkicho	32	22	54
			Sino Funamura	31	23	54
**Total**	3	3	8	185	126	311

### 2.3 Pre-testing

Before actual data collection, pre-testing was conducted in Kofale Woreda in the West Arsi zone, close to the study area. 10 farmers (6 male and 4 female) were interviewed during pre-testing, which allowed enumerators to verify the correct local words and phrases of some of the complex ideas into which enumerators wanted to gain insights. The reliability of the data (questionnaires) was tested by using Cronbach's alpha coefficient.The Cronbach's alpha coefficient was 0.896, indicating that the questionnaires were consistent and reliable [37].

### 2.4 Data analyses

Data was analyzed using descriptive statistics and econometric models. Descriptive statistics such as percentage and mean are used to present a level of knowledge, attitude, and practices towards food safety among male and female food handlers. Moreover, correlation analysis was undertaken to check the association between food safety knowledge, attitude, and practices. Econometrics models such as binary logit, multinomial logit, and ordered probit were used to examine factors associated with level of knowledge, attitude, and practice towards fruit and vegetable safety among male and female food handlers in the study area. The binary logit model was used to analyze binary dependent variables, while multinomial logit was used to analyze categorical dependent variables. The ordered probit was used to analyze naturally ordered categorical dependent variables to examine factors affecting male and female attitude towards fruit and vegetable handling practices. This was done by STATA software version 17.

## 3 Results and discussion

### 3.1 Socio-demographic characteristics of the sampled households

Table 2 summarizes the statistics of the socio-demographic characteristics. Out of the total sampled respondents, the male and female represent 59.49% and 40.51%, respectively. With respect to the gender of the respondent household head, the results show that the proportion of male heads (63.02%) is significantly higher than their female (36.98%) counterparts. Regarding the education level of the respondents, about 12.86% were illiterate, with a higher illiteracy rate (20.63%) among female respondents. About 21.54% and 32.48% attended primary (1st – 4th grade) and full primary (5th – 8th grade) education, respectively. The remaining 28.62% and 4.50% attended secondary (9th – 12th) and higher (>12) education, respectively. The results show that males had a higher attendance rate than females in secondary education (35.10% vs. 19.06%) and in higher education (7.57% vs. 0.00%). The differences in educational status between male and female respondents were statistically significant at 1%. Previous studies indicated that there is an increase in knowledge about food safety as educational status escalates [18].

**Table 2:** Summary statistics of the socio-demographic characteristics of the sampled households.

Variables		Total (%)	Male (%)	Female (%)	test-statistics
Relationship in the household	Male head	63.02	94.60	16.67	
	Female head	36.98	5.40	83.33	3.86**
Education level of respondent	No education	12.86	7.57	20.63	
	Primary education (1st – 4th grade)	21.54	16.21	29.36	
	Full primary education (5th – 8th grade)	32.48	33.51	30.95	10.89***
	Secondary education (9th – 12th)	28.62	35.10	19.06	
	Higher education (>12 grade)	4.50	7.57	0.00	
Age of the respondent in years		37.56	39.30	35.07	3.25*
Family size of respondent		7.13	7.20	7.00	
Number of males (>15 years)		2.02	2.03	2.00	
Number of females (>15 years)		1.90	1.90	1.86	
Number of children (≤15 years)		3.21	3.27	3.14	
Farming experiences of respondent in years		20.86	22.45	18.54	2.03*
Number of observations (n)		311	185	126	

Note: ***, ** and * indicates significant at 1%, 5% and 10% respectively.

The average age of the respondents was 37.56 years with a lower average (35.07) in female respondents, which is significant at 10%. The average family size was 7.13, of which the adult (above 15 years) males and females constitute 2.02 and 1.90, respectively. The rest, 3.21, was constituted by children below or equal to 15 years. The average years in farming was 20.86 with less farming experience in female respondents, which is significant at 10%.

### 3.2 Food safety knowledge towards fruit and vegetables and associated factors

Table 3 presents respondent knowledge towards production, processing, and handling practices of fruit and vegetable and associated factors. Of the total surveyed respondents, 62% knew about the benefits of producing fruit and vegetables at their home garden for consumption, with a higher percentage in the female group. Results also suggest that education level had a positive and significant effect on respondents (i.e., male, female, and female living in the female-headed household) knowledge towards benefits of producing fruit and vegetables in their home garden. The education effect on knowledge level of female respondents (Coef. 0.050, P-value 0.001) were stronger than their male (Coef. 0.312, P-value 0.040) counterparts. These results are consistent with a finding by Ref. [32] in 21 European countries, who noted that being female and having a high education were associated with increased consumption of fruit and vegetables. A study by Ref. [26] in Ethiopia also noted that individuals with higher levels of nutrition knowledge were more likely to accept diversified and healthy foods such as fruit and vegetable. The [10] in Ghana and [9] in Saudi Arabia found a positive significant association between education level and food safety knowledge. Our findings agree with these observations. About 75% of respondents were aware of how to produce fruit and vegetables in their home garden. The results of logistic regression suggest that education level and farming experience had positive and significant associations with the respondent's knowledge on fruit and vegetable production techniques. Our finding agrees with a study by Ref. [38], who noted that education may be an effective tool to increase food safety knowledge among food handlers and thus improve food safety practices.

**Table 3:** Knowledge level towards fruit and vegetable production, processing and storage among male and female food handlers and associated factors.

Producing fruit and vegetable	**Total (%)**	**Male (%)**	**Female (%)**	Male head (coefficient)		Female head (coefficient)		Age (coefficient)		Education level (coefficient)		Farm experience (coefficient)		
				Male	Female	Male	Female	Male	Female	Male	Female	Male	Female	
**Do you know about the benefits of producing fruit and vegetables in the home garden for consumption? (1 = yes)**^**a**^	72.00	66.00	74.00	–	–	–	0.012**	–	–	0.312**	0.050***	–	–	
**Do you know how to produce fruit and vegetable in the home garden? (1 = yes)**^**a**^	75.00	79.00	73.00	–	–	–	–	–	–	0.010*	0.414***	0.203**	0.640***	
Processing and preservation														
**Do you know how to process fresh fruit and vegetable? (1 = yes)**^**a**^	71.00	62.82	69.73	–	0.002**	–	0.140*	–	–	0.071*	0.211*	–	–	
**Do you know how to preserve fresh fruit and vegetable? (1 = yes)**^**a**^	63.60	62.16	65.71	–	–	–	0.200**	–	–	0.091*	0.066**	–	–	
Safe handling and storage														
**Do you know how to handle and store fresh fruit and vegetable? (1 = yes)**^**a**^	73.31	682.43	76.60	–	0.021*	–	0.555**	–	–	–	–	0.270**	0.038**	
**What should you do before eating raw fruit and vegetable?**^**b**^	Wash them with clean water	77.11	76.21	78.41	–	–	–	–	–	–	0.218**	0.007***	0.811**	0.172**
	Clean by hand or piece of cloth	20.64	21.08	20.00	–	–	–	–	–	–	–	–	–	–
	Rub it with ash(Ref)	10.93	11.08	9.79	–	–	–	–	–	–	–	–	–	–
Number of observations	311	185	126	185	126	185	126	185	126	185	126	185	126	

Note: ^a^Parameters were estimated from binary logit regression. ^b^Parameters were estimated from multinomial logit regression. ^(Ref)^ Reference group in the multinomial logit model. Age, education level and farming experience are described continuous variables in the model. ***, ** and * indicates significant at 1%.

5%and 10% probability level respectively. Coefficients of only significant variables are reported in the table.

About 71% and 63.60% of respondents had knowledge about processing and preservation of fresh fruit and vegetables, respectively. Female respondents had higher levels of knowledge about processing (69.73% > 62.82%) and preservation (65.71% > 62.16%) than their male counterparts. Results suggest that the gender of female and men and their educational status had positive and significant effects on their knowledge towards processing and preservation of fruit and vegetables. A recent study by Ref. [31] in Ethiopia noted that fruit and vegetable production in the small farming systems often takes place in home gardens. The studies by [[39], [40], [41]] noted that women who opt to produce different types or varieties that are mainly used for home consumption are in control of home gardens in Ethiopia. Moreover [31], noted that, in the fruit and vegetable value chain, women participate more in processing and preservation activities than their male counterparts. Thus, the present findings agree with these observations.

Out of total respondents, 73.31% have knowledge about how to handle and store fresh fruit and vegetables, with a higher level of knowledge (76.60% > 68.43%) in the female group. The results suggest that gender of females and farming experience had a positive and significant association with knowledge level towards handling and storage techniques of fruit and vegetables in the study area.

Respondents also asked about what you should do before eating raw fruit and vegetables, 77% responded that they wash it with clean water while around 20.64% responded that they clean it by hand or piece of cloth. The results from the multinomial logit model suggest that education status and farming experience had a positive and significant effect on the probability that respondents washing with clean water (rubbing it with ash being the base category for comparison).

### 3.3 Food safety attitude towards fruit and vegetables and associated factors

Table 4 presents the level of respondent's attitude towards fruit and vegetable production, processing and storage, and associated factors. A slight majority of the respondents (40.19%) think they are not likely to produce fruit and vegetables in the home garden, while 54.02% think that they are likely to produce fruit and vegetables in the home garden for consumption. Gender of female, age (only female) and education level had positive and significant associations with the probability of producing fruit and vegetables in the home garden compared to the probability of not producing. About 46.95% of the respondents had a very serious attitude towards producing fruit and vegetables in the home garden for home consumption, while 40.05% had a serious attitude towards producing fruit and vegetables. Gender of male had a positive association with the probability of having a serious attitude, while gender of female had a positive association with the probability of having a very serious attitude towards producing fruit and vegetables in their home garden for home consumption. These results confirm the findings of previous studies suggesting women are more responsible for family consumption than men [31,39,42,43] in Ethiopia and the developing world.

**Table 4:** Attitudes toward fruit and vegetable safety and its associated factors.

Attitude towards producing fruit and vegetable		Total (%)	Male (%)	Female (%)	Male head (coefficient)^a^		Female head (coefficient)^a^		Age (coefficient)^a^		Education level (coefficient)^a^		farm experience (coefficient)^a^	
					Male	Female	Male	Female	Male	Female	Male	Female	Male	Female
**How likely do you think you are producing fruit and vegetable in the garden for your home consumption?**	Not likely	40.19	37.84	43.65	–	–	–	–	–	–	–	–	–	–
	Likely^b^	54.02	56.75	50.00	–	0.111**	–	0.301**	–	0.021**	0.001*	0.120**	–	–
	More likely	5.79	5.40	6.14	–	–	–	–	–	–	–	–	–	–
**How serious do you think producing fruit and vegetable in the home garden for consumption?**	Not serious^b^	8.68	14.59	9.52	–	–	–	–	–	–	–	–	–	–
	Serious	44.05	38.38	52.38	0.001**	–	–	–	–	–	–	–	–	–
	Very serious	46.95	53.51	37.30	–	0.714**	–	0.303**	–	–	–	–	–	–
**How good do you think it is to produce fruit and vegetable in the home garden for consumption?**	Not good^b^	12.00	25.00	8.00	–	–	–	–	–	–	–	–	–	–
	Good	20.58	20.00	21.43	–	–	–	–	–	–	–	–	–	–
	Very good	67.42	65.00	70.57	0.207*	0.111*	–	0.421**	–	–	0.390*	0.488**	–	–
**How difficult is it for you to produce fruit and vegetable in the home garden?**	Not difficult	65.92	65.94	65.87	–	–	–	–	–	–	–	0.200*	0.201*	0.660**
	Difficult^b^	25.72	25.40	26.20	–	–	–	–	–		–	–	–	–
	Very difficult	8.36	8.64	7.74	–	–	–	–	–	–	–	–	–	–
**How confident do you feel in producing fruit and vegetable in the home garden?**	Not confident	6.43	5.94	7.14	–	–	–	–	–	–	–	–	–	–
	Less confident^b^	26.37	24.32	29.36	–	–	–	–	–	–	–	–	–	–
	Confident	55.95	57.30	53.97	–	–	–	–	–	–	–	–	–	–
	More confident	11.25	12.43	9.52	–	–	–	–	–	–	–	–	–	–
Processing and preservation														
**How likely do you think you are processing fresh fruit and vegetable for your consumption?**	Not likely^b^	26.05	27.57	23.81	–	–	–	–	–	–	–	–	–	–
	Likely	69.45	68.65	70.63	–	–	–	–	–	–	–	–	–	–
	More likely	2.89	2.70	3.17	–	–	–	–	–	–	–	–	–	–
**How likely do you think you are preserving fresh fruit and vegetable for consumption?**	Not likely^b^	36.66	34.60	39.68				–	–	–	–	–	–	–
	Likely	59.81	62.70	55.55	–	–	–	–	–	–	0.111*	0.030*	–	–
	More likely	2.25	1.62	3.17	–	–	–	–	–	–	–	–	–	–
**How serious do you think processing fresh fruit and vegetables for your consumption?**	Not serious^b^	27.65	29.95	30.15	–	–	–	–	–	–	–	–	–	
	Serious	44.37	43.78	45.24	–	–	0.021**	–	–	–	0.330**	0.012**	–	–
	Very serious	27.01	29.73	23.01	–	–	–	–	–	–	–	–	–	–
**How good do you think it is to process from fruit and vegetable for consumption?**	Not good^b^	18.97	19.46	18.25	–	–	–	–	–	–	–	–	–	–
	Good	36.33	39.46	31.75	–	–	–	–	–	–	–	–	–	–
	Very good	44.05	40.54	49.20	–	–	–	–	–	–	–	–	–	–
**How difficult is it for you to process (clean or make juice) fresh fruit and vegetable?**	Not difficult^b^	48.23	41.62	57.94	–	–	–	–	–	–	–	–	–	–
	Difficult	31.51	33.51	28.57	–	–	–	–	–	–	–	–	–	–
	Very difficult	18.33	22.16	12.70	–	–	–	–	–	–	–	–	–	–
**Handling and storage**														
**How likely do you think it is that you can get sick from eating spoiled food?**	Not likely^b^	63.67	59.46	69.84	–	–	–	–	–	–	–	–	–	–
	Likely	29.90	33.51	24.60	–	–	–	–	–	–	–	–	–	–
	More likely	5.79	6.48	5.76	–	–	–	–	–	–	–	–	–	–
**How serious do you think it is to be sick from eating spoiled food?**	Not serious^b^	13.05	12.97	18.55	–	–	–	–	–	–	–	–	–	
**How good do you think it is to keep fresh meat, fruit, vegetable or cooked food in a cool place?**	Serious	59.49	62.70	54.76	0.801**	–	–	–	–	–	0.222**	0.031***	–	–
	Very serious	25.08	24.32	26.20	–	–	–	–	–	–	–	–	–	–
	Not good^b^	11.00	13.00	11.90	–	–	–	–	–	–	–	–	–	–
	Good	51.77	52.43	50.79	–	–	–	–	–	–	–	–	–	–
	Very good	36.33	35.67	37.30	–	–	–	–	–	–	0.001*	0.020**	0.111*	0.041**
**How difficult is it for you to keep fruit and vegetable in a cool place?**	Not difficult^b^	30.55	25.40	25.40	–	–	–	–	–	–	–	–	–	–
	Difficult	25.72	25.95	38.09	–	–	–	–	–	–	–	–	–	–
	Very difficult	43.73	24.86	36.50	–	–	–	0.321***	–	0.001*	−0.210*	−0.921**	–	–
**How difficult is it for you to wash fruit and vegetable with clean water?**	Not difficult^b^	94.21	92.97	96.03	–	–	–	–	–	–	–	–	–	–
	Difficult	4.50	5.40	3.17	–	–	–	–	–	–	–	–	–	–
	Very difficult	1.29	1.62	0.79	–	–	–	–	–	–	–	–	–	–
**How difficult is it for you to separate raw food from cooked food?**	Not difficult^b^	93.25	91.89	95.24	–	–	–	–	–	–	–	–	–	–
	Difficult	6.11	7.56	3.97	–	–	–	–	–	–	–	–	–	–
	Very difficult	0.64	0.54	0.79	–	–	–	–	–	–	–	–	–	–

Note: ^a^Parameters were estimated from ordered probit regression. ^b^Base category in the mode estimation. Age, education level and farming experience are described continuous variables in the model. ***, ** and * indicates significant at 1%, 5%and 10% probability level respectively. Coefficients of only significant variables are reported in the table.

Approximately 67% of survey respondents (65% of men and 70.57% of women) had a very good attitude to producing fruit and vegetables in the home garden for consumption and 20.58% had a good attitude. Gender of male-headed households, females in a female-headed household and an education level had positive associations with the probability of having a very good attitude towards producing fruit and vegetables in the home garden. A majority (65.92%) of the respondents stated that it is not difficult for them to produce fruit and vegetables in the home garden, while 25.72% stated that it is difficult for them to produce fruit and vegetables in the home garden. Ordered probit results indicate that female education and farming experience had positive associations with the probability of having a not difficult attitude towards producing fruit and vegetables in the home garden. About 60% of the respondent feels confident in producing fruit and vegetables in the home garden, while 25% feel less confident. However, there is no significant association between the probability of feeling confident in producing fruit and vegetables and gender related factors.

Most of the respondents (69.45%) think that they would likely process fresh fruit and vegetables for home consumption, while 26.05% think that they would not likely process fresh fruit and vegetables for home consumption. This study did not find a significant association between respondent attitude and socio-economic factors towards processing fresh fruit and vegetables for home consumption.

Regarding preservation, 59.81% of the respondents think that they would likely preserve fresh fruit and vegetables for home consumption, while 36.66% think that they would not likely preserve fresh fruit and vegetables for home consumptions. Results suggest that the gender of female and education has significant association with the probability that a respondent would likely preserve fresh fruit and vegetables for home consumption. About 44.37% and 27.65% of the respondents had a serious and not serious attitude towards processing fresh fruit and vegetables for home consumption, respectively. About 27.01% had a very serious attitude towards preservation of fruit and vegetables for home consumption. 44.05% of survey respondents (40.54% of men and 49.20% of women) had a very good attitude towards processing fruit and vegetables for consumption and 36.33% had a good attitude. 48.23% of the respondents stated that it is not difficult for them to process fresh fruit and vegetables. 31.51% stated difficult for their household to process fresh fruit and vegetables. Only 18.33% stated it is very difficult for their household to process fresh fruit and vegetables.

About 63.67% and 29.90% of respondents were not likely to and likely to think that they could get sick from eating spoiled food, respectively. About 5.79% were more likely to think that about the probability of being sick from eating spoiled food. A slight majority of respondents (59.49%) had a serious attitude to being sick from eating spoiled food, while 25.08% had a very serious attitude.

Results suggest that the gender of male and educational status have a significant effect on the probability that the respondent had a serious attitude towards getting sick from eating spoiled food. About 52% of the respondents had a good attitude to keep fresh or cooked foods in a cool box or in the refrigerator, while 36.33% had a very good attitude. About 48% of the respondents stated that it is very difficult for them to keep fresh food in a cool place or refrigerator, while 30.55% said it is not difficult for them. About 28% noted it is difficult. Most of the respondents (94.21%) stated that it is not difficult for them to wash fruit and vegetables with clean water; only 4.50% stated it was difficult. Most of the respondents (93.25%) stated it is not difficult for them to separate raw foods from cooked foods.

### 3.4 Food safety practice of fruit and vegetable and associated factors

Table 5 presents respondents’ production, processing, and handling/storage practices of fruit and vegetable and associated factors. Although more than 70% and 75% surveyed respondents know about the benefits and methods of fruit and vegetables production in the home garden, respectively, all of them did not produce fruit and vegetables in the home garden in the last three months prior to this survey. Of total respondents, about 63% (62.43% of men and 66.50% of women) were produced in the last three months. Consuming fruits and vegetables grown in the home garden is much safer than consuming from market. Gender of females, education and farming experience were positively associated with the probability that they would produce fruit and vegetables in their home garden, while age had a negative effect on fruit and vegetable production.

**Table 5:** Fruit and vegetable production, processing, preservation and handling practices and associated factors.

Practices on producing fruit and vegetable		**Total (%)**	**Male (%)**	**Female (%)**	**Male headed (coefficient)**^**a**^		**Female headed (coefficient)**^**a**^		**Age (coefficient)**^**a**^		**Education (coefficient)**^**a**^		**Experience (coefficient)**^**a**^	
					Male	Female	Male	Female	Male	Female	Male	Female	Male	Female
**Did you produce fruit and vegetables in the home garden in the last three months? (1 = yes)**		63.03	62.43	66.50		–	–	0.210**	−0.210**	−0.002**	0.106*	0.920***	–	0.012**
Processing and preservation														
**Did you process fresh fruit and vegetable at home? (1 = yes)**		73.28	73.78	72.53	–	–	–	–	–	–	–	–	–	–
**Did you preserve fresh fruit and vegetable at home? (1 = yes)**		69.13	66.48	70.01	–	–	–	–	–	–	–	0.712**	0.09**	0.230**
Safe handling and storing														
**Did you clean the kitchen surfaces after preparing foods, yesterday? (1 = yes)**		99.36	99.46	99.20	–	–	–	–	–	–	–	–	–	–
**1. Did you wash hand before starting food preparation? (1 = yes)**		100.00	100.00	100.00	–	–	–	–	–	–	–	–	–	–
**How does you store perishable food such as raw meat, fruit and vegetable?**	In the refrigerator (1 = yes)	33.12	30.81	38.02	–	–	–	–	–	–	–	–	0.091*	0.003**
	Covered (protected from insects & pests) (1 = yes)	73.31	76.21	66.00	–	–	–	–	–	–	–	–	–	–
	Separated from cooked food (1 = yes)	38.91	37.30	41.03	–	–	–	–	–	–	–	–	–	–
	Dry them (1 = yes)	79.74	81.08	77.12	–	–	–	–	–	–	–	–	–	
**Did you wash fresh fruit and vegetable before eating? (1 = Yes)**		78.71	67.84	80.00	–	–	–	–	0.302*	0.012*	0.440*	0.502**	–	–

Note: ^a^Parameters were estimated from binary logit regression. Age, education level and farming experience are described continuous variables in the model. ***, ** and * indicates significant at 1%, 5%and 10% probability level respectively. Coefficients of only significant variables are reported in the table.

Results also show that about 73.28% of the respondents did process fresh fruits and vegetables at home. While 69.13% did preserve fresh fruit and vegetables at home. Education (only female) and years in farming had a positive association with the probability that the respondents would preserve fruits and vegetables at their home. Results also show that about 99.36% of respondents did clean their kitchen surface after cooking food. About 78.71% of the respondents stated that they wash fresh fruit and vegetables before eating. Age and education level had a positive association with the probability that respondents would wash fresh fruits and vegetables before eating.

### 3.5 Correlation among knowledge, attitude and practice of food safety

A summary of the correlation between the level of knowledge, attitude, and practices towards fruit and vegetables is represented in Table 6. No correlation could be obtained between knowledge and practices for the total respondents. However, knowledge had a positive but weak significant association with practices in the female group. The total result implies that even though respondents had some level of knowledge, they still continued to consume unhealthy diets. This finding agrees with a study by Ref. [44], who found an insignificant association between knowledge and practices towards food safety. However, we found a significant positive correlation between knowledge with attitudes (rho = 0.003, p < 0.001 for total, rho = 0.107, p < 0.004 for male rho = 0.000, p < 0.0001 for female) and attitude with practice (rho = 0.009, p < 0.030 for total, rho = 0.010, p < 0.080 for male, and rho = 0.017, p < 0.030 for female). Nevertheless, these correlations were positive and significant, but the correlation between attitude and practice was not very strong in the male group. These findings indicated that food safety knowledge of fruit and vegetable handlers will influence their attitudes and the positive attitude would influence the practices in safe handling of fruit and vegetables. These results agree with other studies which found significant positive correlation between knowledge and attitude by [9,44,45]. Also, a study by Ref. [26] found that nutrition knowledge was positively associated with healthy eating attitudes and practices.

**Table 6:** Correlation among knowledge, attitude and practice level of respondents.

Level	Total (n = 311)		Male (n = 185)		Female (n = 126)	
	Spearman's rho	p-value	Spearman's rho	p-value	Spearman's rho	p-value
Knowledge - Attitude	0.003^a^	0.001	0.107^a^	0.004	0.000^a^	0.001
Knowledge – Practice	0.074	0.156	−0.100	0.209	0.035*	0.040
Attitude – Practice	0.009**	0.030	0.010*	0.080	0.017**	0.030

a , ** and * indicates significant at 1%, 5%and 10% respectively.

## 4 Conclusion

This study aimed to determine the level of knowledge, attitude and handling practice towards the safety of fruit and vegetables and associated factors among male and female food handlers in rural Ethiopia. Education has a positive significant effect on the knowledge, attitude and handling practices of fruit and vegetable handlers. However, the effect was higher in the females. As per this study, food safety knowledge had no association with food safety practice. Although both male and female surveyed household respondents know about the benefits and methods of producing, processing and preserving fruit and vegetables, some of them did not produce, process, and preserve fruit and vegetables. This implied that, in the study area, people didn't translate food safety knowledge into practice. It therefore may require more targeted campaigns (i.e. from awareness creation to behavior change) to increase the ability of the community members to adopt best practices while reducing the barriers associated with consuming unhealthy diets. In-situ training on the basics of food safety shall be implemented to improve the knowledge, attitude, and handling practices of food safety.

We suggest that future studies need to use large-scale data to study the knowledge, attitude, and handling practice towards the safety of fruit and vegetables and associated factors among male and female food handlers in rural Ethiopia. This is in recognition that gender-related factors vary from region to region in Ethiopia.

## Author contribution statement

Girma Gezimu Gebre: Conceived and designed the experiments; performed the experiments; analyzed and interpreted the data; contributed reagents, materials, analysis tools or data; and wrote the paper.

Tibebu Legesse: Analyzed and interpreted the data; contributed reagents, materials, analysis tools or data; and wrote the paper.

Asmiro Abeje Fikadu: Analyzed and interpreted the data; contributed reagents, materials, analysis tools or data; and wrote the paper.

## Funding information

Not Applicable.

## Availability of data and materials

Authors do not have the right to share the data. However, it will be made available to the reader upon request.

## Declaration of competing interest

The authors declare that they have no known competing financial interests or personal relationships that could have appeared to influence the work reported in this paper.
